# Selective embolization of a renal angiomyolipoma using *n*-butyl-2-cyanoacrylate‒lipiodol‒iopamidol mixture under microballoon occlusion resulting in tumor reduction and preservation of normal renal blood flow: A case report

**DOI:** 10.1016/j.radcr.2025.11.070

**Published:** 2025-12-22

**Authors:** Ryuta Okuhira, Nobuyuki Higashino, Tetsuo Sonomura, Maria Kojima, Akira Ikoma, Hiroki Minamiguchi

**Affiliations:** Department of Radiology, Wakayama Medical University, Wakayama City, Wakayama 641-8510, Japan.

**Keywords:** Embolization, Angiomyolipoma, n-Butyl-2-cyanoacrylate, Balloon catheter

## Abstract

A renal angiomyolipoma associated with sporadic lymphangioleiomyomatosis was found in the left kidney of a 30-year-old woman. The tumor had a maximum diameter of 56 mm, and there was a 5 mm diameter aneurysm within the tumor. Embolization using *n*-butyl-2-cyanoacrylate (NBCA)‒Lipiodol‒iopamidol mixtures was planned to prevent the tumor from rupturing. Under microballoon occlusion, NBCA:Lipiodol:iopamidol (1:4:1) mixture was injected into the peripheral part of the tumor and NBCA:Lipiodol:iopamidol (2:3:1) mixture was injected into the proximal part. Embolization completely occluded the feeding arteries of the tumor, and normal renal blood flow was preserved. Computed tomography performed 15 weeks after embolization showed that the embolized branches were completely occluded and normal renal blood flow was maintained. The maximum diameter of the tumor had decreased to 39 mm.

## Introduction

*n*-Butyl-2-cyanoacrylate (NBCA)‒Lipiodol‒iopamidol mixture (NLI) is a liquid embolic material with low adhesiveness [1]. Consequently, it can be used in combination with a balloon catheter, which allows the operator to control the embolic range and prevent migration of the embolic material [1-3]. Renal angiomyolipomas (AMLs) are candidates for embolization to prevent their rupture, depending on the diameters of the tumor and concomitant aneurysm [4,5]. When embolizing an AML, it is important to embolize all tumor vessels, including the aneurysms, and to preserve normal renal blood flow. We report the first case of a renal AML that was successfully treated by selective embolization using NLI under microballoon occlusion.

## Case report

**Fig. 1:**
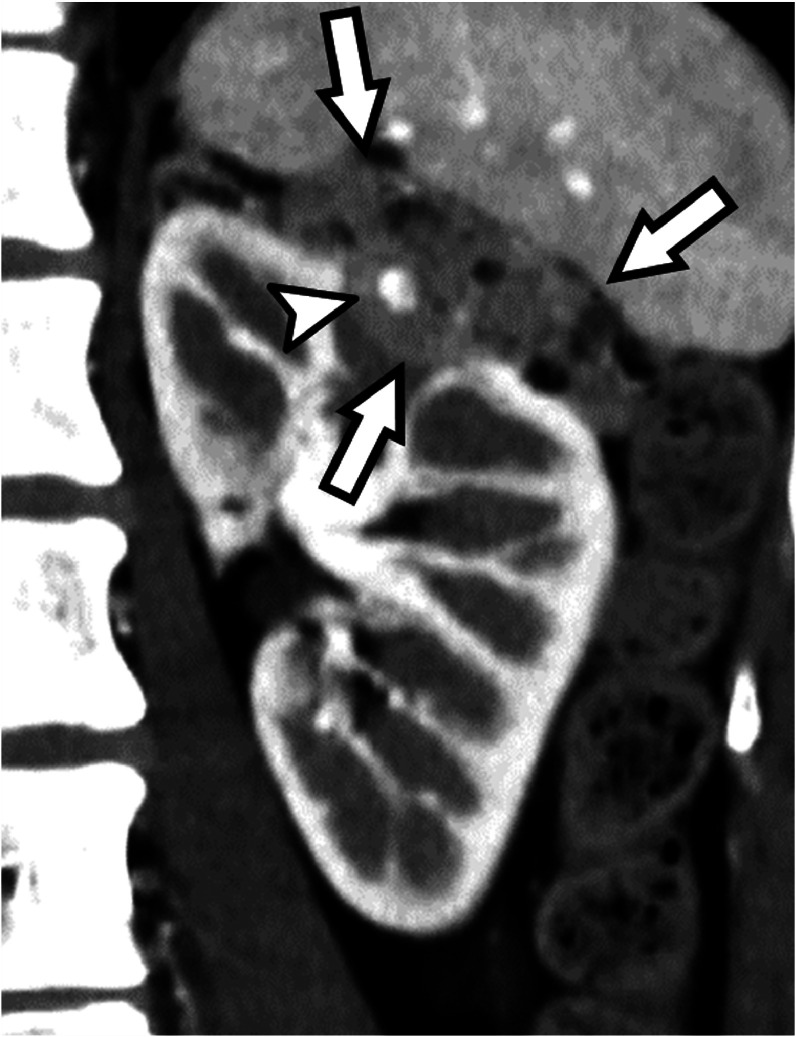
Pre-embolization contrast-enhanced computed tomography image shows a renal angiomyolipoma (arrows) with a maximum diameter of 56 mm in the left kidney and an internal aneurysm (arrowheads).

A 30-year-old woman presented to her primary physician with dyspnea and was diagnosed with a severe left pneumothorax following chest radiography. She was referred to our hospital, where a chest tube was inserted. Computed tomography (CT) of the chest revealed multiple thin-walled rounded empty cysts in both lungs. She subsequently underwent partial resection of the left upper lobe, which was considered the cause of the pneumothorax, and the pathological examination confirmed lymphangioleiomyomatosis (LAM). One year prior to visiting our hospital, she experienced chest pain and dyspnea during childbirth. Three months before presentation, she developed cough and dyspnea, and she was treated for pneumonia by her primary doctor. She had no history of smoking or alcohol consumption, and no significant past medical history, surgical history, or family history. She had been taking oral sirolimus (2 mg/d), an mTOR inhibitor, since the diagnosis of sporadic LAM. Abdominal CT was performed to search for complications and revealed a renal AML with a maximum diameter of 56 mm in the left kidney and a 5 mm diameter aneurysm within the tumor (Fig. 1). The patient was referred to our department for embolization to prevent bleeding from the tumor. Pre-intervention laboratory results included the following: white blood cell count 4.87 G/L; hemoglobin 111 g/L; hematocrit 0.358; platelet count 248 G/L; prothrombin time% 97.8%; activated partial thromboplastin time 27.3 s; serum creatinine 0.58 mg/dL; and C-reactive protein (CRP) 0.14 mg/dL.

**Fig. 2:**
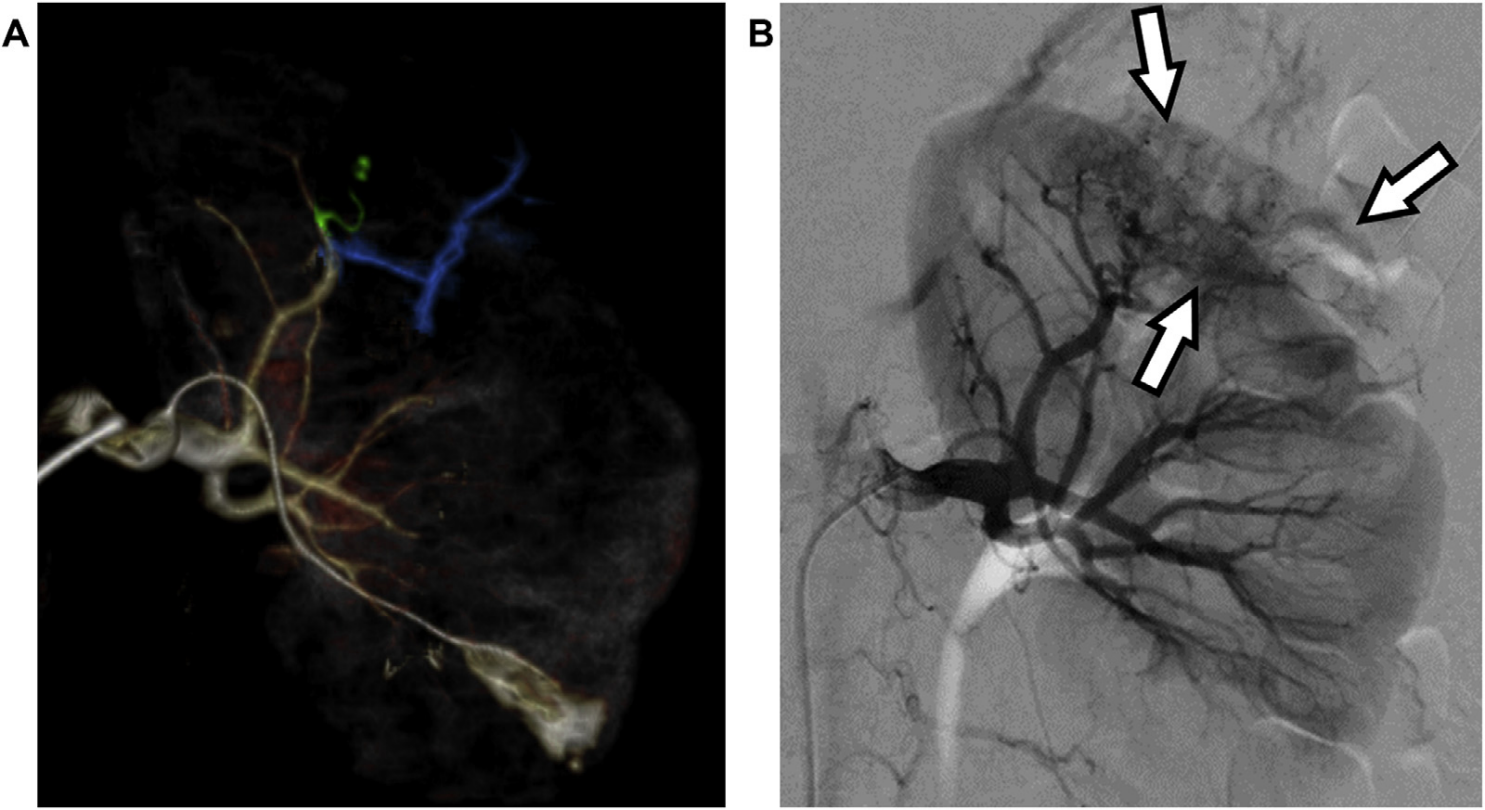
(A) VR image created from computed tomography-arteriography shows the feeding artery of the tumor (blue + yellowish green) and the branch contiguous to the aneurysm (yellowish green). (B) Pre-embolization left renal arteriography image shows the renal angiomyolipoma (arrows) and thin meandering feeding vessels inside the mass.

**Fig. 3:**
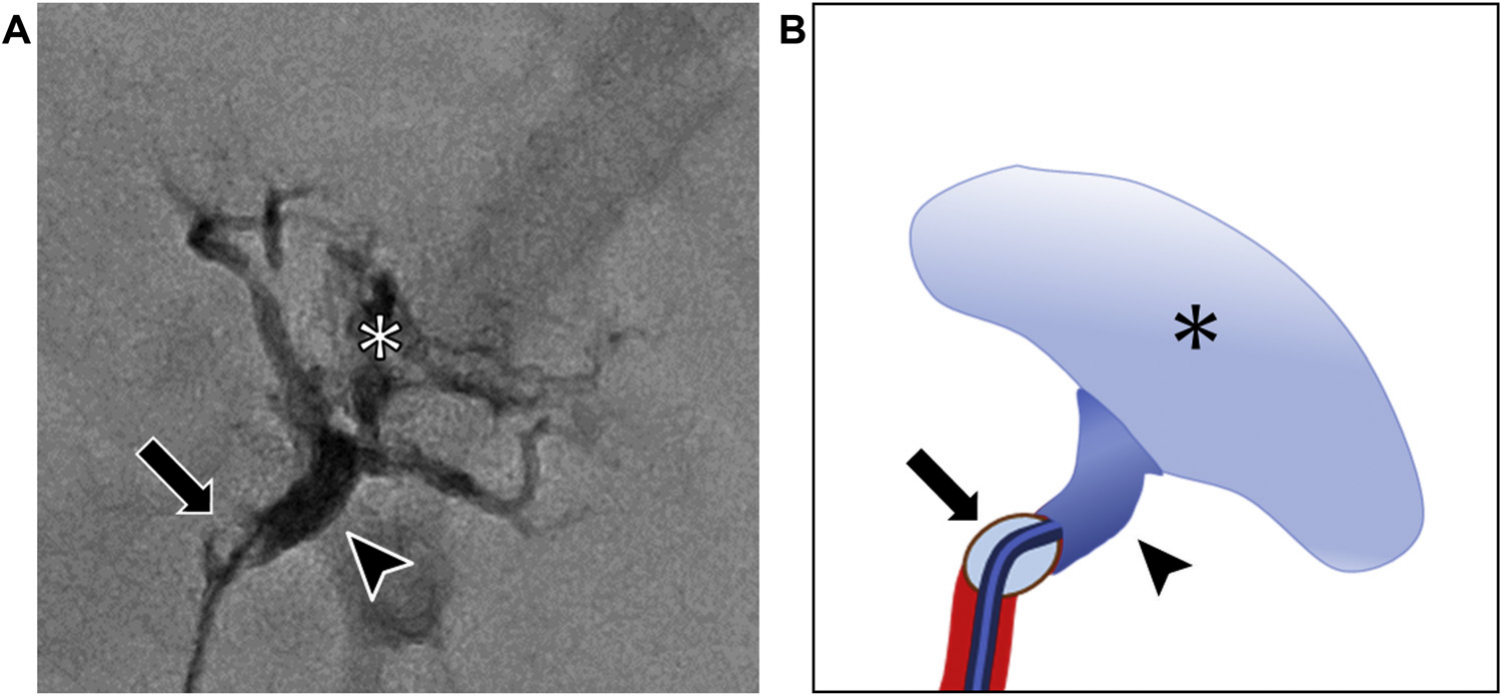
(A) Fluoroscopic image during treatment showing NLI 141 (*) injected into the peripheral region and NLI 231 (arrowhead) injected into the central region with assistance of a microballoon catheter. (B) Schema of embolization using NLI.

**Fig. 4:**
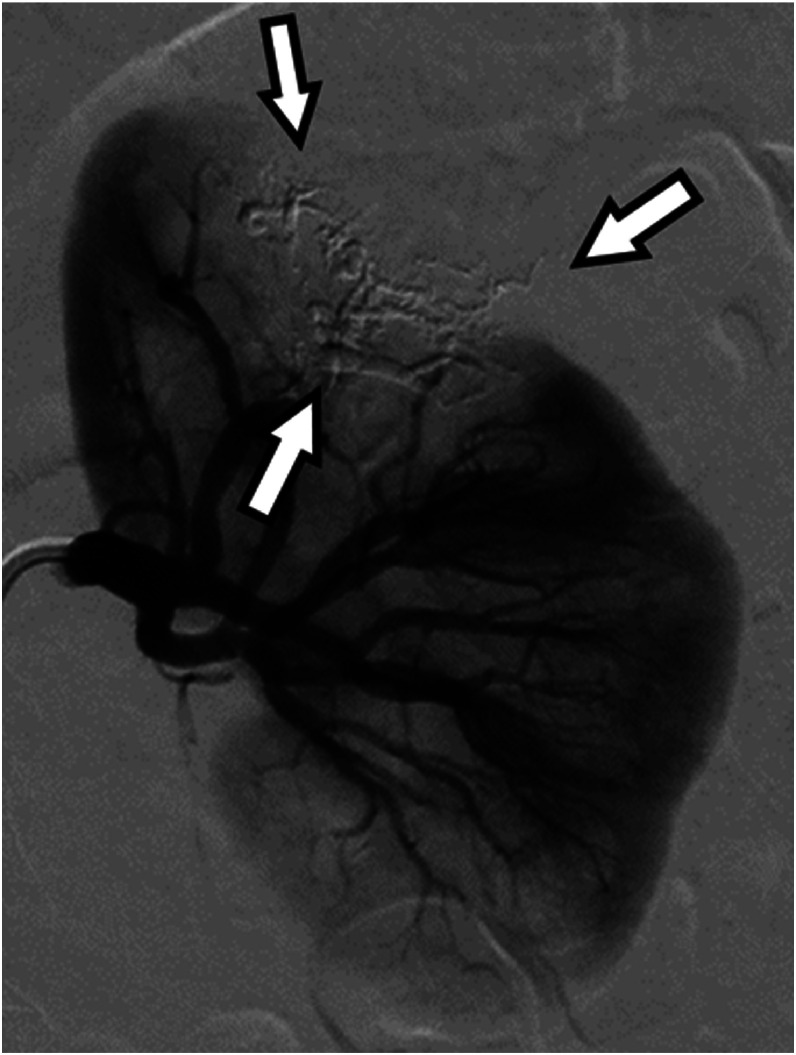
Left renal arteriography immediately after embolization shows the cast-like NLI in the tumor vessels (arrows), the absence of a contrast effect on the tumor vessels, and preservation of normal renal blood flow.

**Fig. 5:**
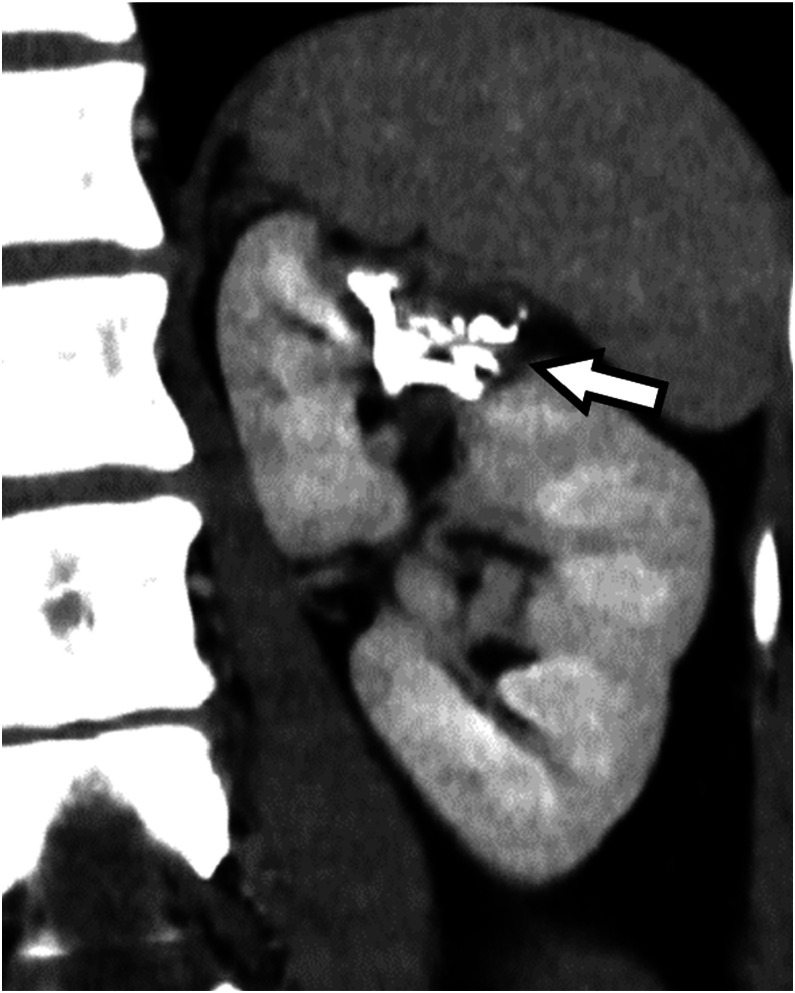
Contrast-enhanced computed tomography image 15 weeks after embolization shows tumor shrinkage, absence of the contrast effect of the tumor, and good contrast effect of the normal renal region. The injected NLI remains in the tumor vessels (arrow).

Angiography was performed for diagnosis and treatment. A 4 Fr sheath (Terumo, Tokyo, Japan) was inserted through the right common femoral artery. CT during arteriography and selective angiography identified the feeding arteries of the tumor (Fig. 2). The feeding branch from the superior division of the left renal artery was highly tortuous and disorganized. Embolization was planned using NLI mixtures under microballoon occlusion to embolize the feeding arteries, including the aneurysm, and to preserve normal renal blood flow. A guiding sheath (Parent Plus 45; Medikit, Tokyo, Japan) was inserted into the left renal artery. A high-flow microballoon catheter (Pinnacle Blue 27; Tokai Medical Products, Aichi, Japan) was inserted across the normal renal artery branch. A coaxial microcatheter (Carnelian MARVEL Non Taper; Tokai Medical Products) was advanced to the distal part of the feeding artery. The NLI mixtures were prepared by mixing NBCA into a mixture of Lipiodol and iopamidol using a pumping method with 2.5 mL syringes and a 3-way stopcock. The pumping frequency was 15 reciprocations in 15 seconds. The final ratios of NBCA:Lipiodol:iopamidol were 1:4:1 (NLI141) and 2:3:1 (NLI231). Under microballoon occlusion, 0.8 mL of NLI141 was injected into the peripheral parts of the feeding artery via a coaxial microcatheter, after which 0.5 mL of NLI231 was injected into the proximal parts of the feeding artery (Fig. 3). Three minutes later, the balloon was deflated and the microballoon catheter was removed from the artery. Angiography immediately after embolization showed complete occlusion of the feeding artery and preservation of normal renal blood flow (Fig. 4). After the embolization, the patient developed left-sided abdominal pain and a low-grade fever, both of which were manageable with antipyretic analgesics and resolved spontaneously. Laboratory tests revealed an elevated CRP level, peaking at 13.28 mg/dL, without other signs of infection. Her renal function remained stable. She was discharged 3 days after the procedure. CT performed 15 weeks after embolization showed that the embolized branch was disrupted and normal renal blood flow was maintained (Fig. 5). The maximum diameter of the tumor had decreased to 39 mm.

## Discussion

Embolization is considered for renal AML when the tumor diameter exceeds 4 cm or the aneurysm within the tumor exceeds 5 mm [4-6]. Because embolization is often performed at a young age, complete embolization of the tumor vessels and preservation of normal renal blood flow (to preserve renal function) are necessary. There is no consensus on the optimal embolic material, although polyvinyl alcohol particles, microspheres, liquid embolic materials (eg, NBCA‒Lipiodol mixture [NL] and ethanol), metallic coils, or combinations of these materials have been used for embolization [[7], [8], [9], [10], [11]]. Liquid embolic materials are suitable because they can reach peripheral vessels, achieving high tumor shrinkage and low re-treatment rates [12].

This is the first report of embolization using NLI for a renal AML. NLI is characterized by low adhesiveness and compatibility with balloon catheter systems, offering precise control of its distribution [[1], [2], [3]]. NLI infusion under microballoon occlusion allows complete embolization of the target vessel and strict control of the embolic extent [13]. In the present case, the triaxial microballoon system, which comprised a high‑flow microballoon catheter and a coaxial microcatheter, enabled accurate delivery of NLI while protecting the adjacent normal branches, resulting in complete embolization of the tumor vessels and the aneurysm. According to the maximum tumor diameter, the tumor reduction rate in this case was 30%, suggesting that embolization using NLI may achieve a reduction in tumor size similar to that achieved using other liquid embolic materials [12].

Previous animal studies have shown that NLI141 disperses into the distal vasculature, whereas NLI231 forms a more cohesive cast within the proximal portion of the vessel [3]. Combining these formulations enabled distal penetration and central consolidation in this case.

Further studies are needed to evaluate the safety and efficacy of NLI for the embolization of AML. Additionally, further research is required to determine the optimal mixing ratio of NLI used for embolization.

## Conclusion

Selective embolization of a renal AML using NLI under microballoon occlusion may be an effective and safe treatment because it allows reliable embolization of the tumor vessels and preservation of the normal vessels.

## Patient consent

The patient provided informed consent for preparation and publication of this case report.
